# Education Research: High-Fidelity Simulation to Evaluate Diagnostic Reasoning Reveals Failure to Detect Viral Encephalitis in Medical Trainees

**DOI:** 10.1212/NE9.0000000000200020

**Published:** 2022-11-17

**Authors:** Melissa B. Pergakis, Wan-Tsu W. Chang, Camilo A. Gutierrez, Benjamin Neustein, Jamie E. Podell, Gunjan Parikh, Neeraj Badjatia, Melissa Motta, David P. Lerner, Nicholas A. Morris

**Affiliations:** From the Department of Neurology (M.B.P., C.A.G., J.E.P., G.P., N.B., M.M., N.A.M.), Program in Trauma (M.B.P., W.-T.W.C., B.N., J.E.P., G.P., N.B., M.M., N.A.M.), and Department of Emergency Medicine (W.-T.W.C.), University of Maryland School of Medicine, Baltimore, MD; and Department of Neurology (D.P.L.), Lahey Hospital and Medical Center, Burlington, MA.

## Abstract

**Background and Objectives:**

Delays in treatment of both herpes simplex virus (HSV) encephalitis and seizures are associated with poor patient outcomes, but many physicians fail to recognize HSV despite classic presenting symptoms. Our goal was to assess trainee performance in a simulation-based case to recognize HSV encephalitis as the underlying etiology of refractory status epilepticus.

**Methods:**

This is a prospective, observational, single-center simulation-based study of participants ranging from subinterns to attending physicians managing a patient with viral encephalitis complicated by nonconvulsive status epilepticus. Using a modified Delphi approach, we developed a list of critical actions. The primary outcome measure was critical action item sum score. We compared level of training and performance using analysis of variance as validity evidence to support our findings.

**Results:**

Fifty-nine trainees completed the simulation. The mean sum of critical actions completed was 13.9/25 (56%). Eighty percent of trainees administered an appropriately dosed benzodiazepine, and 97% administered a second-line agent. Despite 88% of trainees obtaining a lumbar puncture, only 47% recognized viral encephalitis as the most likely diagnosis with 36% starting appropriate treatment. There was significant effect of training level on critical action sum score (level 1 mean score [SD] = 10.8 [1.5] vs level 2 mean score [SD] = 12.2 [2.5] vs level 3 mean score [SD] = 13.9 [3.0] vs level 4 mean score [SD] = 18.2 [3.2], *p* < 0.001, *R*^2^ = 0.38).

**Discussion:**

Although initial seizure treatment was sufficient, failure to recognize HSV encephalitis was common with few trainees initiating appropriate treatment potentially leading to poor outcomes in real-life scenarios. High-fidelity simulation holds promise as an assessment tool in identifying trainee knowledge gaps and why classic clinical cases escape trainee diagnosis.

Herpes simplex virus (HSV) encephalitis is the most common cause of fatal sporadic encephalitis in the United States accounting for 10%–20% of annual viral encephalitis cases presenting to the hospital.^1^ In untreated HSV encephalitis, mortality reaches 70%, and in patients receiving appropriate treatment, mortality remains between 20% and 30%.^1-3^ Patients typically present with fever, altered level of consciousness, and seizures. Unfortunately, many physicians may fail to consider HSV encephalitis on the differential diagnosis of patients presenting with classic symptoms. One study found that only 29% of patients ultimately diagnosed with HSV encephalitis received treatment in the emergency department.^4^

Seizures are seen in up to one-half of patients presenting with HSV encephalitis.^5^ A review of status epilepticus management at academic medical centers found substantial performance gaps between recommended practice and “real-world” treatment patterns.^6^ Simulation-based assessments of status epilepticus management have corroborated those findings with physicians frequently underdosing benzodiazepines and only 57% adding a second-line agent.^7^ Some have proposed that simulation-based assessments can assist program directors in evaluating trainees' level of epilepsy milestone achievement.^8^ Previous studies have focused on adherence to seizure cessation algorithms at the expense of viewing status epilepticus as a symptom of potentially serious neurologic disease requiring diagnosis and treatment. By contrast, most patients with status epilepticus have no history of epilepsy,^9^ and many will harbor acute pathologies requiring emergent care.^10^ These findings suggest that a comprehensive assessment of status epilepticus management should evaluate both seizure management and comprehensive workup for and management of acute treatable causes.

We assessed trainee performance in managing a simulated case of status epilepticus due to HSV encephalitis. We hypothesized that trainees may fail to make a canonical diagnosis because of the need to concurrently manage the acute symptom of status epilepticus. We also sought validity evidence of our simulation as a measure of competency in trainees.

## Methods

### Setting and Study Design

This is a prospective, single-center simulation study performed between February 2018 and January 2022. It was conducted by 3 neurointensivists (M.B.P., W.-T.W.C., N.A.M.). Each simulation was captured by video for review by the raters. Participants ranging from neurology subinterns to attending physicians voluntarily participated individually in the study. They did not receive any information about the case before the simulation. Before participation, they completed a questionnaire regarding demographics, level of training, and prior experience with the management of multiple neurologic emergencies including meningoencephalitis and status epilepticus. Participants completed a pretest regarding the management of neurologic emergencies, including but not limited to meningoencephalitis and status epilepticus. They were allowed to use smartphones if they wished during the simulation. The simulation took between 20 and 30 minutes to complete. After the simulation clinical scenario was completed, they debriefed with a neurointensivist for an additional 20–30 minutes. The debriefing was structured following the Debriefing with Good Judgment approach to invoke reflective practice leading to behavioral change and performance improvement.^11^ Simulation took place on an afternoon when trainees were rotating in the neurocritical care unit. They were excused from clinical duties, and consequently, the simulation took place within their duty hours. We told participants that their performance did not have any effect on their grade or evaluation for the rotation. They did not receive any financial compensation for participation in the study.

### Clinical Simulation Case and Trainee Assessment Development

We previously published development of the clinical simulation case and critical actions checklist using a modified Delphi method.^12^ The clinical case, critical actions checklist, and rating scale can be found in the supplementary material of our prior publication.^12^ We chose the items on the critical action checklist based on the Neurocritical Care Society's Emergency Neurologic Life Support protocols, cross-referenced with guidelines from the Epilepsy Foundation/American Epilepsy Society,^13^ the Infectious Disease Society of America,^14,15^ and the Neurocritical Care Society.^16^ A board-certified neurologist with certification in neurocritical care (N.A.M.), an additional board-certified neurologist with certification in neurocritical care at a different institution (D.P.L.), and a board-certified neurologist with board certification in epilepsy and clinical neurophysiology (C.A.G.) reviewed the clinical scenario and checklist. The final list checklist represented a consensus among the three.

### Simulator and Simulation Environment

In a prebriefing, the simulation operator oriented the participants to the simulation environment and SimMan. We asked them to verbalize any components of the neurologic examination they wished to perform and to verbalize any orders or diagnostics. Diagnostics including laboratory results, neuroimaging, and EEG findings were displayed on a monitor in the simulation room. If participants wished to place a consult, they did so through telephone request in the simulation room. The simulation operator acted as the consultant. Consultant responses were not scripted; however, consistency of response was ensured through intermittent viewing of cases for standardization. If they were able to independently perform a procedure such as intubation, we asked them to perform that skill, otherwise they were asked to consult a service to perform any procedure outside of their skill set. A nurse embedded participant accompanied participants through the entirety of the simulation scenario. The nurse had direct communication with the simulation operator using an earpiece.

We used the SimMan 3G manikin (Laerdal, Wappinger Falls, NY). SimMan 3G can depict both neurologic and physiologic signs including pupillary constriction, voice through an internal speaker, respiratory patterns, and seizures. Although SimMan is not able to perform the entirety and nuances of a neurologic examination, participants can obtain these findings by asking the nurse embedded participant at bedside whether extraocular movements, the motor examination, and reflexes are intact. A bedside monitor displays telemetry, arterial blood pressure, and oxygen saturation. These parameters can be altered by the simulation operators.

The simulation room represented a patient room on a medical/surgical floor. It included all equipment needed to perform the clinical scenario. Medications included IV fluids, rapid sequence intubation medications, benzodiazepines, antiseizure medications, anesthetic drips, thiamine, and dextrose. Although medications were available in the simulation room, they were not visible to participants. Airway equipment included nasal cannula, non-rebreather mask, bag valve mask, laryngoscope, endotracheal tubes (ETTs), Bougie, and a ventilator. Other equipment included a lumbar puncture tray and EEG machine.

### Clinical Scenario

Participants performed individually by responding to a rapid response on a medical/surgical floor for a 54-year-old woman with a history of alcohol/heroin use and remote seizures who was prescribed levetiracetam who was initially brought into the emergency department for altered mental status. Per report from her boyfriend, she “hadn't been herself” for several days. She complained of headaches, reported continued alcohol and drug use, and denied missing doses of levetiracetam. The nurse at bedside witnessed the patient have a generalized tonic-clonic seizure for about 1 minute followed by somnolence. The patient's medical history also included hypertension, diabetes mellitus, and remote history of seizure. Her medications included lisinopril, metformin, and levetiracetam 500 mg twice daily.

The initial phase of the case required participants to appropriately workup altered mental status with seizure. During the initial workup, participants were expected to gather histories for seizures and alcohol and drug use disorders. They were expected to check the patient's temperature (she was febrile) and evaluate for meningismus. If obtained, lumbar puncture showed lymphocytic pleocytosis with xanthochromia indicative of viral encephalitis. EEG showed right temporal discharges evolving into seizures. As the case progresses, the patient suffered recurrent seizures and ultimately refractory status epilepticus requiring intubation as well as escalation of antiseizure medications to anesthetic infusion for seizure control. Successful management of the case required recognition that the patient had an underlying meningoencephalitis, recognition of both convulsive seizures and nonconvulsive status epilepticus, appropriate treatment of presumed bacterial and viral meningoencephalitis, appropriate dosing of antiseizure medications and anesthetic infusions for status epilepticus, and identifying basic EEG characteristics for status epilepticus and burst suppression.

### Outcomes

The primary outcome was the sum score of 25 critical action items performed by trainees only (Table 2). When actions required completion of a proximal action (for instance, administration of acyclovir required consideration of a diagnosis of viral encephalitis), participants were not credited for either action. Empirical treatment of a condition was taken as evidence of consideration of a diagnosis. The secondary outcome was a global rating scale of trainee performance ranging from 1 to 5 with 5 indicating superior performance. Critical actions were rated by 2 independent coders, M.B.P. and N.A.M. (both certified in neurology and neurocritical care) using videotaped recordings of each participant's simulation. Both coders were aware of participants' level of training.

### Validity Evidence

We previously provided validity evidence for the development of the cases and checklist action items in the domains of content evidence and response process, according to Messick's framework.^12^ We aimed to provide further validity evidence to support our findings in the domains of content evidence, internal structure, and relationship to other variables.^17^ After simulation, participants completed a questionnaire indicating how realistic they felt the scenario to be along with their level of engagement using a 7-point Likert scale ranging from “not at all” to “extremely” to provide content evidence. For internal structure, we calculated inter-rater reliability of both sum scores of critical action items and global rating scales. Regarding relationship with other variables, we assessed sum score of critical action items by level of training, training background, self-rated experience, and multichoice pretest performance assessing knowledge of general neurology, neurocritical care, and general critical care principles. We grouped the critical action items into 3 categories: diagnostic workup critical actions, assessment and interpretation critical actions, and management critical actions. Diagnostic workup critical actions included ordering intracranial imaging, reviewing complete blood count and basic metabolic panel, obtaining point of care glucose, ordering a toxicology screen and levetiracetam level, obtaining blood cultures, lumbar puncture, and EEG. Assessment critical action items included verbalizing a differential diagnosis, recognizing viral encephalitis as the most likely diagnosis based on cerebrospinal fluid results, and recognizing nonconvulsive seizures on EEG without assistance. Management critical action items included administering thiamine, appropriately dosed benzodiazepine, appropriately dosed second-line agent, dexamethasone, and empiric antibiotics and antivirals, recognizing a need for intubation at the appropriate time, preoxygenating before intubation, confirming ETT placement, and starting an anesthetic drip at the appropriate time at proper dosing and titrating the anesthetic drip until either seizure cessation or burst suppression.

We stratified participants into 4 levels to compare level of training. Level 1 included neurology subinterns and neurosurgery interns (who are not involved in consults for altered mental status and management of status epilepticus and lack any critical care training). Level 2 participants included postgraduate year (PGY)-2 neurology residents (who routinely perform consults for altered mental status, seizure, and status epilepticus at our institution), surgical critical care fellows, and medicine-trained critical care fellows (who average 1 month of neurocritical care experience). Level 3 participants included PGY-3 and PGY-4 neurology residents (who have 2 or more years of experience performing altered mental status, seizure, and status epilepticus consults as well as 2 or more months of experience in the neurocritical care unit) and emergency medicine-trained critical care fellows (who have training in the initial management of patients presenting with altered mental status, seizure, and status epilepticus and have at least 1 month experience in the neurocritical care unit). Level 4 participants included neurocritical care fellows and attending physicians in neurocritical care (who were all board-certified in neurology).

We evaluated performance on critical action items relating to airway management including preoxygenation, intubating the patient at the correct time in the clinical scenario, and assessing ETT placement using chest x-ray between participants with critical care training and those without. We hypothesized that participants with critical care training would perform better on these items. We also evaluated performance between participants with a training background in neurology and those without a training background in neurology on status epilepticus-related action items including administration of appropriately dosed benzodiazepine, administration of an appropriately dosed second-line agent, initiation of an anesthetic drip, recognition of nonconvulsive status epilepticus on continuous video EEG, and titration of the anesthetic drip to seizure control or burst suppression. We hypothesized that participants with training in neurology would perform better on status epilepticus-related action items.

### Statistical Analysis

We reported descriptive statistics as mean (SD) for continuous variables and counts and frequencies for categorical variables. Based on a previous study in which we found trainees to perform 68% of critical action items,^18^ we felt adequately powered with a sample size of 59 trainees (1 − β = 0.8, α = 0.05). We performed an analysis of variance for univariate comparisons between participants' levels of training with post hoc comparison between training levels using the Scheffe test given unequal sample sizes between groups and the increased sensitivity of this post hoc test for comparisons involving more than 2 means. We measured effect size using the coefficient of determination, *R*^2^. Using a *t* test, we compared performance of participants trained in critical care and those without critical care training on airway-related critical action items. We also used a *t* test to compare performance of participants with a background in neurology and those without a background in neurology on status epilepticus-related critical action items. Using Pearson correlation, we assessed participants' performance with self-rated experience and performance on a subset of questions focused on meningoencephalitis and status epilepticus from a written, multiple-choice test testing diagnosis and management of all neurologic emergencies as well as self-rated experience. Agreement among the raters was assessed using the intraclass correlation (ICC) with an ICC value of greater than 0.75 considered as excellent. The results were considered statistically significant if the *p* < 0.05.

All analyses were performed using IBM SPSS Statistics 27. The reporting format is in accordance with the guidelines established by the Strengthening the Reporting of Observational Studies in Epidemiology study and the extended guidelines for health care simulation research.^19,20^

### Standard Protocol Approvals, Registration, and Patient Consents

This study was approved by the University of Maryland Institutional Review Board, which waived the need for informed consent.

### Data Availability Statement

On reasonable request, the data that support the findings of this study are available from the corresponding author, N.A.M.

## Results

Sixty-two participants completed the simulation including 59 trainees and 3 neurocritical care attending physicians (Table 1). All invited trainees agreed to participate.

**Table 1:** Characteristics of Participants

Age, y, mean (SD)	32.3 (4.5)
Female, n (%)	26 (42)
Level of training, n (%)	
Neurology subintern	2 (3)
Neurosurgery intern	2 (3)
PGY-2 neurology resident	18 (29)
PGY-3 neurology resident	6 (10)
PGY-4 neurology resident	5 (8)
Medical critical care fellow	8 (13)
Emergency medicine critical care fellow	8 (13)
Surgical critical care fellow	1 (2)
Neurocritical care fellow	9 (15)
Neurocritical care attending physician	3 (5)
Primary work location, n (%)	
Medical intensive care unit	13 (21)
Surgical intensive care unit	2 (3)
Neurocritical care unit	14 (23)
Neurology floor	30 (48)
ENLS certification, n (%)	31 (50)
Experience in medical simulation, n (%)	54 (87)

Abbreviations: ENLS = Emergency Neurological Life Support; PGY = postgraduate year.

### Primary and Secondary Outcomes

The mean sum of critical action items completed by trainees (excluding attending physicians) was 13.9 of a total of 25 (56%) (Table 2). The mean (SD) global rating scale for trainees was 3.2 (0.95) of 5. Eighty percent (47/59) of trainees administered a benzodiazepine at appropriate dosing and 97% (57/59) administered a second-line antiseizure medication appropriately dosed. Only 69% (41/59) recognized status epilepticus on continuous EEG, and 63% (37/59) of trainees started an anesthetic drip. Despite 73% (43/59) of trainees obtaining a lumbar puncture, only 47% (28/59) recognized viral encephalitis as the most likely diagnosis with 36% (21/59) starting appropriate treatment. For status epilepticus-related critical actions, the mean sum score for trainees was 6.8 of 11 (62%). For meningoencephalitis-related critical actions, the mean sum score for trainees was 3.3 of 7 (46%).

**Table 2:** Trainees' Performance of Critical Action Items

Examination and history-taking critical actions, n (%)	
Assess for meningismus	31 (53)
Assess for fever	49 (83)
Elicit seizure history	43 (73)
Elicit substance abuse history	43 (73)
Diagnostic workup critical actions, n (%)	
Order noncontrast head CT	57 (97)
Review CBC and BMP	58 (98)
Check point of care blood glucose	22 (37)
Order toxicology screen	38 (64)
Order levetiracetam level	13 (22)
Obtain blood cultures	27 (46)
Obtain lumbar puncture	43 (73)
Obtain continuous EEG	54 (92)
Assessment and interpretation critical actions, n (%)	
Verbalize differential diagnosis	32 (54)
Recognize viral encephalitis as most likely diagnosis based on CSF results	28 (47)
Recognize nonconvulsive seizures on EEG without assistance	41 (69)
Management critical actions, n (%)	
Administer thiamine	9 (15)
Administer lorazepam 4 mg for 1 dose	47 (80)
Administer appropriate second-line antiseizure therapy	57 (97)
Administer dexamethasone	9 (15)
Administer empiric vancomycin, ceftriaxone, ampicillin, and acyclovir	21 (36)
Recognize need for intubation	54 (92)
Preoxygenate for intubation	26 (44)
Order CXR and confirm ETT placement	22 (37)
Administer third^-^line treatment with anesthetic drip at proper dosing	37 (63)
Continue to bolus and titrate anesthetic agent to achieve either seizure or burst suppression	42 (71)

Abbreviations: BMP = basic metabolic panel; CBC = complete blood count; CXR = chest x-ray; ETT = endotracheal tube.

### Validity Evidence

#### Response Process

Participants rated the simulation scenario as emotionally engaging (mean score [SD] = 6.1 [0.94]), range 1 (not at all engaging) to 7 (very engaging) and realistic (mean score [SD] = 6.0 [0.85]), range 1 (not at all realistic) to 7 (very realistic).

#### Internal Structure

Inter-rater reliability was excellent with ICCs for sum score of critical action items and global rating scores 0.76 and 0.94, *p* < 0.001, respectively.

#### Relationship to Other Variables

**Figure 1:**
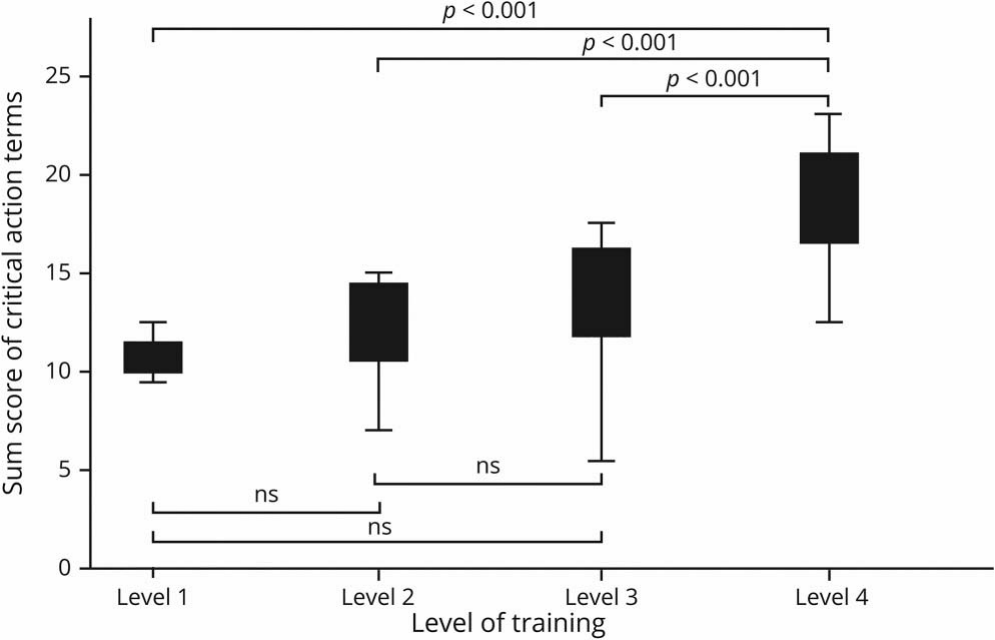
Critical Action Sum Score by Level of Training Effect of level of training on sum score of critical action items. Level 1 mean score (SD) = 10.8 (1.5) vs level 2 mean score (SD) = 12.2 (2.5) vs level 3 mean score (SD) = 13.9 (3.0) vs level 4 mean score (SD) = 18.2 (3.2), *p* < 0.001, *R*^2^ = 0.38.

**Figure 2:**
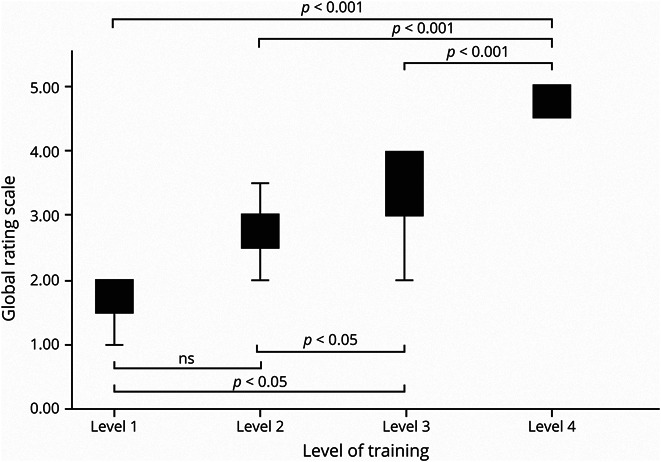
Global Rating Score by Level of Training Effect of level of training on global rating scores. Level 1 mean score (SD) = 1.7 (0.58) vs level 2 mean score (SD) = 2.7 (0.57) vs level 3 mean score (SD) = 3.3 (0.60) vs level 4 mean score (SD) = 4.6 (0.68), *p* < 0.001, *R*^2^ = 0.62.

There was a moderately strong effect of level of training on sum score of critical actions as seen in Figure 1 (level 1 mean score [SD] = 10.8 [1.5] vs level 2 mean score [SD] = 12.2 [2.5] vs level 3 mean score [SD] = 13.9 [3.0] vs level 4 mean score [SD] = 18.2 [3.2], *p* < 0.001, *R*^2^ = 0.38). In addition, there was a strong effect of level of training on the global rating score of performance as seen in Figure 2 (level 1 mean score [SD] = 1.7 [0.58] vs level 2 mean score [SD] = 2.7 [0.57] vs level 3 mean score [SD] = 3.3 [0.60] vs level 4 mean score [SD] = 4.6 [0.68], *p* < 0.001, *R*^2^ = 0.62). There was no effect of level of training on sum score of diagnostic workup critical action items (level 1 mean score [SD] = 4.5 [0.87] vs level 2 mean score [SD] = 4.6 [1.1] vs level 3 mean score [SD] = 5.2 [0.82] vs level 4 mean score [SD] = 5.5 [1.3], *p* = 0.07). However, there was an effect of level of training on sum score of assessment critical action items (level 1 mean score [SD] = 1.3 [1.0] vs level 2 mean score [SD] = 1.0 [0.82] vs level 3 mean score [SD] = 1.5 [0.87] vs level 4 mean score [SD] = 2.5 [0.59], *p* < 0.001, *R*^2^ = 0.27) and management critical action items (level 1 mean score [SD] = 3.2 [1.9] vs level 2 mean score [SD] = 4.6 [1.6] vs level 3 mean score [SD] = 5.1 [1.4] vs level 4 mean score [SD] = 7.0 [1.4], *p* < 0.001, *R*^2^ = 0.30).

The sum scores of critical actions were positively correlated with self-reported experience in meningitis and status epilepticus (*r* = 0.38, *p* < 0.01). However, sum scores did not correlate with performance on a multiple-choice pretest score (*r* = 0.13, *p* = 0.31).

**Figure 3:**
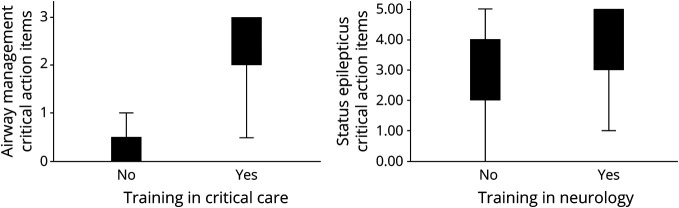
Training-Specific Critical Actions by Training Background (A) Performance on airway-related critical action items by training background. Participants with critical care training outperformed those who were not trained in critical care (mean airway score [SD] = 2.1 [0.99] vs 0.28 [0.60], *p* < 0.001). (B) Performance on status epilepticus-related critical action items by training background. Participants with neurology training outperformed those who were not neurology-trained (mean score of status epilepticus items [SD] = 4.0 [1.2] vs 2.8 [1.5], *p* < 0.05).

Participants who had training in critical care performed better on critical action items related to airway management than those participants who were not trained in critical care (mean airway score [SD] = 2.1 [0.99] vs 0.28 [0.60], *p* < 0.001) as demonstrated in Figure 3A. Participants who had training in neurology performed better on critical action items related to status epilepticus management than those who were not trained in neurology (mean status epilepticus score [SD] = 4.0 [1.2] vs 2.8 [1.5], *p* < 0.05) as seen in Figure 3B.

## Discussion

We assessed trainee performance during a case of status epilepticus caused by viral encephalitis using high-fidelity simulation. Although most trainees successfully performed the first steps of status epilepticus treatment, many failed to identify and treat the underlying etiology. We also showed validity evidence to support our findings such as an effect of level of training on sum scores and global rating scales, an effect of training background on performance of airway-related items and status epilepticus-related items, reported engagement, and a high-level of participant-perceived realism.

Trainees performed well on the initial workup and management of the clinical scenario with most trainees ordering basic serum laboratory tests and head imaging and obtaining a lumbar puncture. In addition, once the patient had a clinical seizure, 80% of trainees properly administered the correct dose of lorazepam, while nearly all administered a second-line antiseizure medication at appropriate dosing and ordered EEG. However, many trainees failed to integrate the information obtained from their initial diagnostic workup to concurrently treat possible underlying etiologies. Less than half of trainees recognized viral encephalitis as the most likely diagnosis based on the CSF profile, in the context of fever, altered mental status, seizures, and prodromal behavioral changes. In addition, the broad differential of a patient presenting with altered mental status seemed to be abandoned entirely. For instance, despite the patient having a history of significant daily alcohol use, few trainees administered thiamine. Our finding echoes prior research showing that thiamine repletion is often forgotten in patients admitted to the intensive care unit with alcohol use disorder.^21^

We did not formally capture debriefing as part of the study to better elucidate why trainees made mistakes, and thus, no qualitative analysis of debriefing took place. Nonetheless, we observed a few common themes. First, we appreciated anchoring bias, whereby participants prematurely settled on a diagnosis such as alcohol withdrawal or medication nonadherence despite information to the contrary (the patient reported alcohol use on the day of presentation and denied missing doses of antiseizure medication). Second, participants reported that the urgency of managing status epilepticus hindered deeper consideration of the underlying diagnosis. Participants reported a substitution bias by which they substituted a more algorithmic solution for a relatively simple problem (seizures) for a less certain solution to the more complex problem of altered mental status. Once status epilepticus was successfully treated, participants still refrained from reconsidering the underlying diagnosis. We hypothesize that partially this error resulted from contextual cues. Participants were told that they were part of a rapid response and may not have accepted responsibility for diagnosing the underlying etiology of the more immediate problem, leaving that duty to the primary team. Future studies should include thematic analysis of debriefing as part of the study to confirm or refute these suppositions.

During debriefing, many trainees, particularly more novice trainees, reported significant stress and anxiety managing the decompensating patient during the simulation encounter which has also been evidenced in other simulated resuscitation scenarios by resident trainees suggesting that cognitive overload may impede performance.^22^ Traditional cognitive learning educational theory suggests that working memory is limited, and when cognitive load is overwhelmed with extraneous factors (such as stress, anxiety, fatigue), learning and transfer of knowledge suffers.^23^ Thus, the goal of education should be to mitigate these factors to optimize learning. However, many of these extraneous, affective factors are intrinsic factors to clinical practice in medicine, especially high-acuity scenarios, so the goal of education and knowledge acquisition in these scenarios is to gradually introduce affective factors during learning encounters as opposed to minimize them.^24^ High-fidelity simulation provides a unique learning environment that tests trainees' clinical knowledge with affective factors (stress, anxiety, fatigue, uncertainty) inherent to clinical practice in a safe environment and challenges traditional models of medical education and trainee assessment. Future studies should further explore the affective factors trainees encounter while providing cognitive strategies to optimize performance during difficult clinical scenarios.

Previous studies have shown nonadherence by both trainees and practicing physicians to status epilepticus guidelines using simulation.^7,8^ We similarly found room for improvement in the proper dosing of benzodiazepines and especially in the proper use of anesthetic agents. While previous studies have tested an algorithmic approach to the treatment of status epilepticus, they have not emphasized diagnostic reasoning.^7,8,25^ In real-world practice, trainees are faced with the management of status epilepticus and a need to diagnose and treat the underlying etiology. Trainees in our study completed 62% of the critical actions related to status epilepticus, but only 46% of critical actions related to the workup and management of meningoencephalitis with only 15% and 36% administering dexamethasone and broad-spectrum antivirals and antibiotics, respectively. Our findings corroborate previous studies showing that delays in acyclovir therapy for HSV encephalitis are common and most frequently due to failure to consider the diagnosis despite highly suggestive clinical features.^26^ Simulation-based mastery learning has shown improved performance in trainees' adherence to the status epilepticus management algorithm.^25^ Our findings suggest that educators should develop tiered simulation-based mastery learning cases of increasing complexity to facilitate comprehensive diagnosis and management of both seizures and underlying etiologies. In addition, debriefing with experienced faculty is an essential part of medical simulation targeted to individual learners' strengths and weaknesses in understanding and elucidating why trainees make medical errors. Through the Debriefing with Good Judgment approach, the facilitator explores the trainee's knowledge, assumptions, and feelings that drive their actions and helps close performance gaps through discussion and targeted instruction. Future research and educational initiatives should focus on evaluating debriefing discussions to elucidate common themes that may generalize across trainees.

Our study suggests that paper and pencil tests may not reflect trainee competence in these complex clinical scenarios as performance on a pretest regarding management of meningoencephalitis and status epilepticus was not correlated with performance during the simulation scenario. This finding further substantiates previous studies comparing performance on written examinations with clinical performance.^27,28^ Performance in a simulation scenario is more likely to represent the critical thinking and decision-making that is required of trainees during real-time clinical encounters in contrast to paper and pencil tests that ask for selection of the best answer. Testing trainees' proficiency in this way often ignores the nuances of an individual patient or clinical scenario and the time it takes to arrive at such decisions. Simulation-based mastery learning offers a way to accelerate progress along Miller's pyramid of assessment by offering clinical experience instead of rote knowledge transfer.^29^

Our study has several limitations. It is a single-center study and may not represent the larger population of trainees. Coders were not blinded to participant background which could have biased the global rating scale. However, given the agreement between the sum score of critical actions and the global rating scale, we feel that this did not influence ratings. In addition, 5 cases were only coded by one rater because of video recording malfunction, but given the high degree of agreement between raters on other cases, we still included these cases in the final analysis. We also did not correlate performance in the simulation scenario with clinical performance although we did provide other forms of validity evidence through Messick's framework.^12^ Future studies should compare performance during simulation with other measures including the in-service examination as well as faculty and milestone assessments of trainees. Finally, future research should delve into the reason why trainees make cognitive errors on classic clinical presentations through the formal qualitative analysis of debriefings.

High-fidelity simulation is a promising assessment tool for the management of meningoencephalitis and status epilepticus. During a simulated case of HSV encephalitis complicated by nonconvulsive status epilepticus, trainees performed well on the initial management of seizures and subsequent status epilepticus but failed to appropriately explore and treat the underlying cause. Our findings warrant further study to understand why classic cases escape trainee diagnosis.

## Appendix Authors

**Table:**

Name	Location	Contribution
Melissa B. Pergakis, MD	Department of Neurology, Program in Trauma, University of Maryland School of Medicine, Baltimore	Design and conceptualized study; analyzed the data; interpreted the data; drafted the manuscript for intellectual content
Wan-Tsu W. Chang, MD	Department of Neurology, Department of Emergency Medicine, and Program in Trauma, University of Maryland School of Medicine, Baltimore	Design and conceptualized study; interpreted the data; revised the manuscript for intellectual content
Camilo A. Gutierrez, MD	Department of Neurology, University of Maryland School of Medicine, Baltimore	Design and conceptualized study; revised the manuscript for intellectual content
Benjamin Neustein, MAT	Program in Trauma, University of Maryland School of Medicine, Baltimore	Major role in acquisition of data
Jamie E. Podell, MD	Department of Neurology, Program in Trauma, University of Maryland School of Medicine, Baltimore	Interpreted the data; revised the manuscript for intellectual content
Gunjan Parikh, MD	Department of Neurology, Program in Trauma, University of Maryland School of Medicine, Baltimore	Interpreted the data; revised the manuscript for intellectual content
Neeraj Badjatia, MD, MS	Department of Neurology, Program in Trauma, University of Maryland School of Medicine, Baltimore	Interpreted the data; revised the manuscript for intellectual content
Melissa Motta, MD	Department of Neurology, Program in Trauma, University of Maryland School of Medicine, Baltimore	Interpreted the data; revised the manuscript for intellectual content
David P. Lerner, MD	Department of Neurology, Lahey Hospital and Medical Center, Burlington, MA	Design and conceptualized study
Nicholas A. Morris, MD	Department of Neurology, Program in Trauma, University of Maryland School of Medicine, Baltimore	Design and conceptualized study; analyzed the data; interpreted the data; drafted the manuscript for intellectual content

## Glossary

ETT: endotracheal tube
HSV: herpes simplex virus
ICC: intraclass correlation
PGY: postgraduate year
